# Molecular evidence of senescence in corneal endothelial cells of senescence-accelerated mice

**Published:** 2009-04-15

**Authors:** Xuan Xiao, Ye Wang, Huaqing Gong, Peng Chen, Lixin Xie

**Affiliations:** 1 Department of Ophthalmology, Renmin Hospital of Wuhan University, Wuhan 430060, China (Ph.D. candidate); 2 State Key Laboratory Cultivation Base, Shandong Provincial Key Laboratory of Ophthalmology, Shandong Eye Institute, Qingdao, China

## Abstract

**Purpose:**

To investigate senescent evidence in corneal endothelial cells (CECs) of the senescence-accelerated mouse (SAM), which is considered a suitable animal model for the further study of the senescent mechanism in CECs.

**Methods:**

Thirty-six male mice from a senescence resistant mouse strain (SAM R1) and a senescence-prone strain (SAM P8) at various ages (1, 6, and 12 months) were analyzed in this study. The endothelial cell density (ECD) and cell viability were detected using trypan blue and alizarin red dyes while the senescent cells were observed by senescence-associated β-galactosidase (SA-β-Gal; pH 6.0) staining. In addition, ultrastructure was observed using an electron microscope. The senescence-related genes (*p16^INK4a^*, *p19^ARF^*, *p21^WAF1/CIP1^*, and *p53*) in the CECs were visualized via immunohistochemistry and were quantitatively detected using real-time polymerase chain reaction (PCR). Signal proteins of phospho-extracellular signal-regulated kinase 1/2 (p-ERK 1/2) were detected by western blot analysis.

**Results:**

Our results indicated that the ECD values decreased with increasing age in both the SAM-R1 and SAM P8 series where the values in the older SAM p8 series decreased even lower than in the older SAM R1 series. The mean decreased rate was 2.276% per month in the SAM R1 and 2.755% per month in the SAM P8 series. In addition, changes in the senescence-like ultrastructure were observed in the CECs of both strains, and the increase in the positive staining of SA-β-Gal was observed in both strains as well. It is worth noting that such changes were more significant in the SAM P8 strain. Immunohistochemical detection assays indicated the expression of p-ERK 1/2, p16^INK4a^, p19^ARF^, p21^WAF1/CIP1^, and p53 (nuclear localization for each) in each age group analyzed. Furthermore, the results of real-time PCR studies showed an increase in the expression of *p16^INK4a^* mRNA as a function of age in the SAM R1 strain and in the early senescence stage of the SAM P8 strain in addition to an increase in the expression of *p21^WAF1/CIP1^* and *p53* mRNA as a function of age in the SAM P8 strain (no significant increase was observed in the SAM R1 strain). Additional results from western blot analysis demonstrated an age-related increase in the quantity of the p-ERK 1/2 proteins in both strains.

**Conclusions:**

The SAM R1 and SAM P8 strains represent suitable models for the study of CEC senescence in vivo. In addition, the progression of cellular senescence in CECs occurs more quickly in the SAM P8 strain as opposed to the SAM R1 strain. Our results also indicate that the p16^INK4a^ signaling pathway may play a key role in the early stages of senescence in CECs while the p53/p21^WAF1/CIP1^ signaling pathway may exert its principle effect in the late stages of senescence in CECs. Further study is still required about the role of the mitogen-activated protein kinase (MAPK) signaling cascade in the process of senescence in CECs.

## Introduction

The corneal endothelial cells (CECs) represent a single layer of cells located between the corneal stroma and the aqueous humor. This thin layer of cells performs important functions such as serving as an ionic pump to maintain corneal transparency and is also known to provide a barrier between the corneal stroma and the aqueous humor. Although some studies have indicated that the human corneal endothelial cells (HCECs) retain the ability to proliferate ex vivo, the HCECs are in fact nonproliferative in vivo, and furthermore, senescence-like changes have been shown to occur in CECs while the endothelial cell density (ECD) has been shown to decrease as a function of increasing age [1-4]. Additional studies have indicated that accidental or surgical trauma in addition to disease may stimulate the ECD value to decrease at a rapid rate [5,6], which may be related to an accelerated progression of senescence in CECs [7,8]. However, the mechanism of the pathological and physiologic senescence process in CECs is still unclear.

Some studies have reported that senescence-related genes such as *p16^INK4a^*, *p21^WAF1/CIP1^*, and *p53* are expressed ex vivo in the CECs from several species [9-12]. However, the aging process may be quite different in vivo due to the influence of the cultural, environmental, and passage stress. Our laboratory has demonstrated that *p16^INK4a^*, *p21^WAF1/CIP1^*, *P27^KIP1^*, and *p53* are all expressed in normal HCECs in vivo, and we conclude from the age-dependent increase in *p16^INK4a^* expression that the p16^INK4a^ signaling pathway may play a key role in the process of senescence in HCECs [8]. However, it is still unclear the molecular mechanism of the aging process of HCECs in vivo. Thus, a suitable animal model is essential for further study.

The senescence-accelerated mouse (SAM) is an appropriate animal model [13,14]. This model consists of accelerated senescence-prone strains (SAM P) and senescence-resistant strains (SAM R). The life span of the SAM P and SAM R strains are approximately 358 days and 526 days, respectively. In addition, the SAM P strains are characterized by an early onset and more rapid advancement of senescence after a normal developmental process whereas the SAM R strains are characterized by a persistent and normal senescence process. They are widely used in the study of the mechanisms responsible for senescence as well as in elucidating the pathogenesis and treatment of age-dependent disorders [15]. Each SAM strain has strain-speciﬁc, age-associated pathological phenotypes [16-24] such as the SAM R1 strain exhibits a decrease in the production of photoreceptors and ganglion cells with an increase in age while the SAM P8 exhibits changes in the retinal pigment epithelium (RPE)-Bruch’s-choriocapillaris complex [25,26]. These two strains have also been studied for brain aging, which is considered coevolution with eyes [27-29] However, to our knowledge, there has been no study describing the changes in the corneal endothelium of the SAM. Thus, we want to verify the existence of senescence in the SAM P8 and SAM R1 strains from the cell morphology and molecular levels and evaluate whether this animal model is appropriate for any further study of the senescent signal pathway in vivo.

## Methods

### Animals

All of the experiments subscribed to the ARVO Statement for the Use of Animals in Ophthalmic and Vision Research. The number of animals used in this study was minimized as was any potential distress or discomfort to the animals in accordance with the aforementioned regulations. All of the mice were male and were divided into two groups known as the SAM P8 (n=18) experimental group and the SAM R1 (n=18) experimental group. Each group was studied at various ages including 1, 6, and 12 months after birth. The animals were obtained from the First Teaching Hospital of Tianjin University of Traditional Chinese Medicine, Tianjin, China.

### Detection of the endothelial cell density, the viability, and the exponential rate of cell loss

In this set of experiments, half of each cornea was cut and stained with trypan blue and alizarin red dyes [8]. Subsequently, five non-adjacent visual fields of the endothelium from the central (as close as possible to the center) and paracentral regions were observed using an optical microscope with an ocular micrometer (Eclipse E800; Nikon, Tokyo, Japan). The ECD and viability values were calculated as the mean value of repeated measurements. The monthly rate of endothelial cell loss was calculated according to the formula reported previously (ECD_2_=ECD_1_e^-rt^) [30,31].

### Electron microscopy

The second half of each corneal section from above was ﬁxed in 4% buffered glutaraldehyde, washed in a buffered solution of 0.2% sucrose-kakodyl for 4−10 h, post-ﬁxed for 1 h in 1% osmium tetroxide, and was dehydrated in graded alcohol concentrations. For analysis involving the use of JEOL JSM-840 scanning electron microscopy (SEM) (JEOL ,Tokyo, Japan), the quarter of specimens were replaced with isoamyl acetate, air-dried, and sputter-coated with gold before examination on the microscope. For analysis involving transmission electron microscopy (TEM), semi-thin sections (1 µm in thickness) of another quarter of specimens were embedded in an epoxy resin for orientation purposes and were subsequently stained with toluidine blue. In addition, ultrathin sections were stained with uranyl acetate-lead citrate and were examined on a Hitachi H-7000 transmission electron microscope (Hitachi, Tokyo, Japan). The central and paracentral regions were also observed.

### Senescence-associated β-galactosidase activity staining

Senescence-associated (SA)-β-galactosidase activity was detected as described previously [8]. For these analyses, half of each cornea was washed with PBS, fixed in 4% formaldehyde, and was incubated with a staining solution consisting of 1 mg/ml X-Gal in dimethylformamide, 5 mM potassium ferrocyanide, 5 mM potassium ferricyanide, 150 mM NaCl, and 2 mM MgCl_2_ all in PBS at pH 6.0 for 12 h at 37 °C.

### Immunohistochemical localization of protein in the senescence signaling pathway in corneal endothelial cells

Half of each cornea was cut, immediately fixed in a solution of 40 g/l formaldehyde, and embedded in paraffin. The paraffin-fixed samples were sliced into 4 μm thick sections and subjected to immunohistochemical staining using the EliVision^TM^ plus kit (Maxim Corp, Fuzhou, China) according to the manufacturer’s protocol. The primary antibodies were the rabbit monoclonal anti-p-ERK 1/2 antibody (1:200; Cell Signaling Technology, Beverly, MA), the mouse monoclonal anti-p16 antibody (1:300; Santa Cruz Biotechnology, Santa Cruz, CA), the rabbit polyclonal anti-p19 antibody (1:300; Abcam, Cambridge, UK), the rabbit polyclonal anti-p21 (1:20; Lab Vision Corp, Fremont, CA), and the rabbit polyclonal anti-p53 antibody (1:50, Lab Vision Corp). A colored reaction was detected using the Diaminobenzidine (DAB) kit (Boster, Wuhan, China). In addition, negative controls for all antigens consisted of tissue sections incubated with PBS instead of a primary antibody. The slides were photographed using a Nikon DXM 1200 digital camera (Nikon, Tokyo, Japan).

### Quantitative real-time polymerase chain reaction detection of the mRNA of senescence-related genes

The corneal tissues obtained from each of the SAM strain groups at various ages were placed on a culture plate under a surgical microscope (Zeiss S4, Jena, Germany) with the endothelium side facing upward. Descemet’s membrane including the endothelium was immediately detached from the underlying stroma using 0.12 mm microforceps. Total RNA was then extracted from the CECs using the RNeasy Mini kit (Qiagen GmbH, Hilden, Germany) according to the manufacturer’s protocol. Transcription of the corresponding cDNA was performed using a cDNA synthesis kit (MBI Fermentas, Heidelberg, Germany) according to the manufacturer’s instructions. All of the polymerase chain reactions (PCRs) were performed using the SYBR green I Mastermix (Tiangen, Beijing, China) in a final volume of 12 µl where the cDNA sample was tested by the ABI Prism 7500 Real Time PCR System (Applied Biosystems, Foster City, CA) according to the manufacturer’s instructions. The expression of genes was normalized to glyceraldehyde-3-phosphate dehydrogenase (*GAPDH*). The primer sequences for these reactions are shown in Table 1.

**Table 1:** Primers used for real-time polymerase chain reaction.

**Gene name**	**Primer sequence**	**Product length**
*P16INK4a*	F:AGGACCCCACTACCTTCTCCC	172 bp
	R:AAATATCGCACGATGTCTTGATG	
*P19ARF*	F:GGCATGAACCGCCGACCTAT	101 bp
	R:GGGCAGGCACAAACACGAAC	
*P21WAF1/CIP1*	F:CCCGAGAACGGTGGAACT	115 bp
	R:TGCAGCAGGGCAGAGGAAG	
*P53*	F:CCCCAGGATGTTGAGGAGTTT	153 bp
	R:TTGAGAAGGGACAAAAGATGACA	
*GAPDH*	F:CTGCCCAGAACATCATCCCT	119 bp
	R:GGTCCTCAGTGTAGCCCAAGA	

### Western blot analysis

The CECs were obtained as mentioned above. Protein was extracted from CECs using the RIPA lysis buffer (50mM Tris PH7.4, 150mM NaCl, 1%Triton X-100, 1% sodium deoxycholate, 0.1%SDS, sodium orthovanadate, and sodium fluoride; Galen, Beijing, China) according to the manufacturer’s instructions. Each of the prepared samples in a final volume of 15 µl (containing a total of 50 µg of protein) were run on a 10% SDS–PAGE gel for 1 h at 160 V and was then transferred to a polyvinylidene difluoride (PVDF) membrane (Millipore, Billerica, MA). The blots were blocked in 5% non-fat dry milk dissolved in TBST ( 20mM Tris PH7.5, 0.5mM NaCl, 0.05%Tween-20) for at least 1 h and were then incubated with the primary antibody in TBST for 1 h at room temperature. The blots were then washed three times with 10 ml samples of TBST and were subsequently incubated for 1 h at room temperature with a horseradish peroxidase-conjugated secondary antibody (Amersham Biosciences, Uppsala, Sweden) in TBST. The blots were washed again three times with 10 ml of TBST. The membranes were then developed with an SuperSignal West Femto Maximum Sensitivity substrate (Pierce Biotechnology, Rockford, IL) and exposed to X-ray film (Kodak, Rochester, NY). Immunoreactive bands were visualized via chemiluminescence and quantified using NIH Image 1.62 software (National Institutes of Health, Bethesda, MD). The primary antibodies were the rabbit monoclonal anti-p-ERK 1/2 antibody and the rabbit anti-Erk1/2 antibody (1:2,000 and 1:1,000, respectively; Cell Signaling Technology).

### Statistical analyses

The statistical differences of each sample upon comparison between different strains and ages groups were analyzed using the two-way analysis of variance (ANOVA) and Student-Newman-Keul's (SNK) test. All p values less than 0.05 were considered to be statistically significant.

## Results

### Endothelial cell density and viability

**Figure 1:**
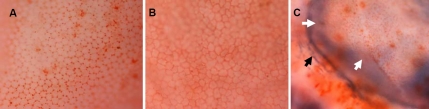
Corneal endothelium of the SAM strains stained with alizarin red and trypan blue for detection of the cell morphology, density and viability. **A**: The endothelium of a one-month-old specimen from the SAM R1 strain is shown. Most of the cells are regular hexagon. Magnification: 400X. **B**: The endothelium of a six-month-old specimen from the SAM P8 strain shows a significant number of polygonal cells. Magnification: 400X. **C**: The endothelium of a 12-month-old specimen from the SAM P8 strain is displayed. The arrows indicate the hyperplasia of the tunica vasculosa on the endothelial surface layer. The covering of the tunica vasculosa causes most of the cells displayed to look unclear. The blue lines (black arrow) indicate the hyperplastic blood vessel. Magnification: 100X.

**Figure 2:**
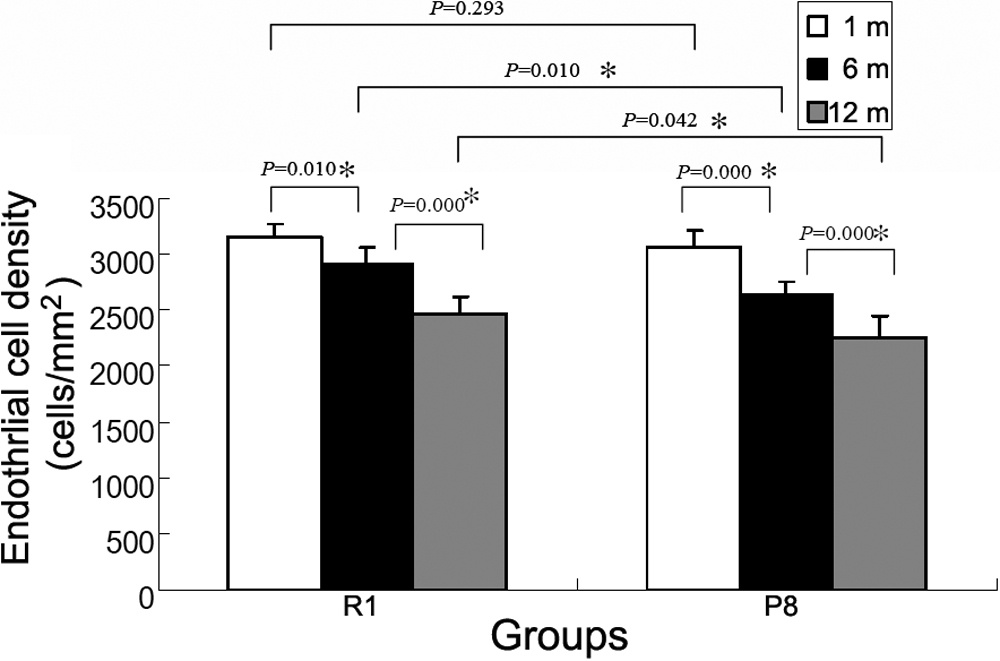
Changes in the endothelial cell density values at various ages for each experimental group. The results indicate a significant decrease in the ECD values of the SAM R1 strain at six months (p=0.010 versus one-month-old specimen) and at 12 months (p=0.000 versus both one- and six–month-old specimens). There is also a significant decrease in the ECD values of the SAM P8 specimens at six months (p=0.000 versus one-month-old specimen) and at 12 months (p=0.000 versus both one- and six-month-old specimens). In addition, there is no significant difference in the ECD values at one month between the SAM R1 and the SAM P8 strains (p=0.293), but there is a significant decrease in the ECD values of both six- and 12–month-old specimens between the SAM R1 and the SAM P8 strains (p=0.010 and p=0.042, respectively). The results indicate the decrease of ECD in SAM P8 is faster than in SAM R1. An asterisks mark a statistically significant difference (p<0.05).

After staining with trypan blue and alizarin red, the intercellular borders stained red while the nuclei of damaged cells stained blue (Figure 1). The ECD decreased in both SAM R1 and P8 strains, but the decline in the P8 strain was faster (Figure 2). The cell viability in each group was higher than 85%, and the exponential rate of cell loss was shown in Table 2. The mean rate of endothelial cell loss (from 1- to 12-month-old) of the SAM R1 strain was 2.276±0.71% per month, while that of the SAM P8 strain was 2.755±0.85% per month.

**Table 2:** Corneal endothelial changes for all experimental subjects.

**Groups (strain, age in months)**	**Number of fields examined^#^**	**ECD (cells/mm^2^)**	**Viability (%)**	**Exponential rate of cell loss (%/m)**
R1 1 m	15	3160±114.02	96.0±2.0	
R1 6 m	15	2900±158.11	92.8±1.48	1.717±1.55*
R1 12 m	15	2460±151.66	92.2±1.30	2.742±1.27**
P8 1 m	15	3060±151.66	93.4±2.30	
P8 6 m	15	2640±114.02	94.4±1.95	2.953±1.31*
P8 12 m	15	2260±181.66	89.0±2.92	2.590±1.77**

In the table, m is the age in months. The hash mark (#) indicates that the number of fields examined came from three individual corneas in each experimental group. An asterisk means the exponential rate of cell loss rate is from the age of one month to the age of six months. The double asterisk means the exponential rate of cell loss rate is from the age of six months to the age of 12 months.

### Ultrastructure

**Figure 3:**
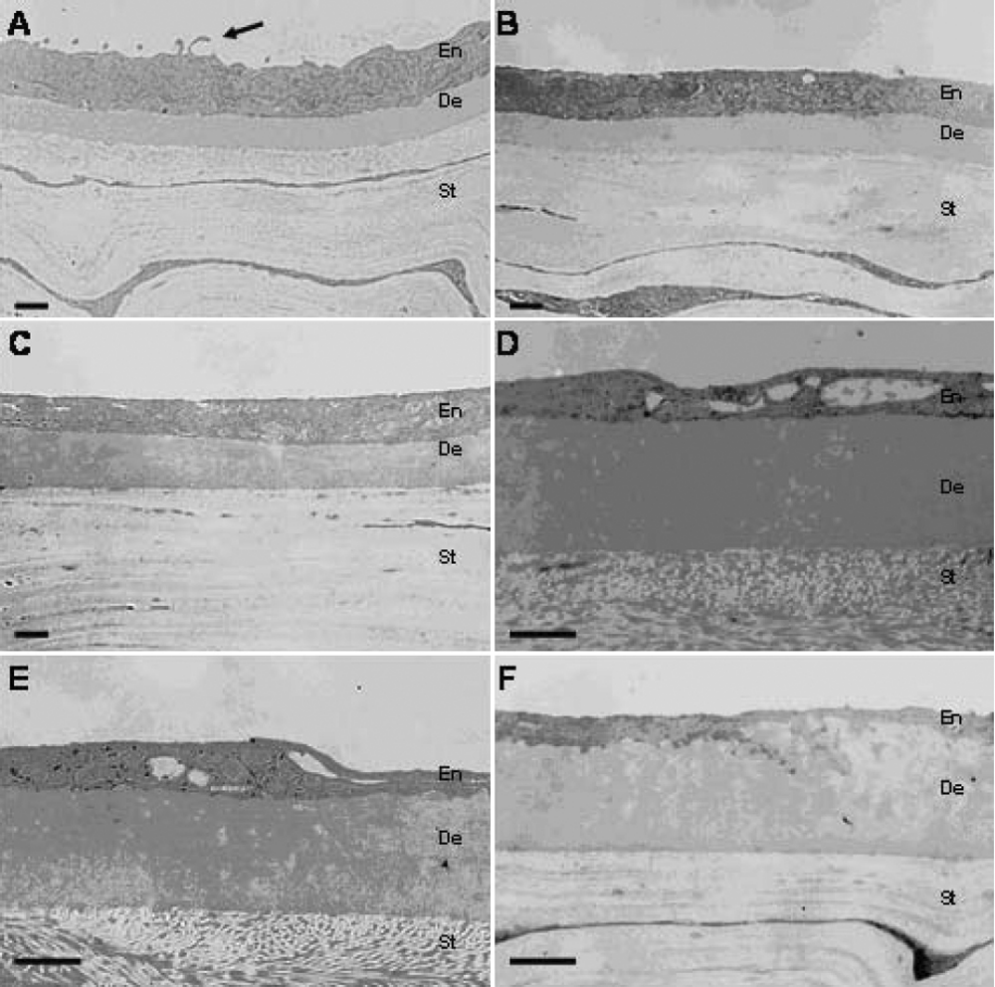
Analysis of transmission electron microscope imaging. The cornea from one-month-old mice of the SAM R1 and the SAM P8 experimental groups are shown (**A** and **B**, respectively). The thickness of the endothelium was greater than or equal to the thickness of the Descemet’s membrane. The microvilli were abundant (locations indicated by arrows). **C**: Analysis of six-month-old specimens from the SAM R1 experimental group shows that the thickness of endothelium was also less than or equal to the thickness of the descemet’s membrane. **D**: Analysis of six-month-old specimens from the SAM P8 experimental group shows that the thickness of the endothelium decreased significantly and that many of the interstices were located in the interlamination. **E**: Analysis of 12-month-old specimens from the SAM R1 experimental group showed that the thickness of the endothelium was uneven and that many of the interstices could be observed. **F**: Analysis of the 12-month-old specimens from the SAM P8 experimental group showed that the thickness of endothelium was very thin. Scale bars: 1 μm. En: endothelium; De: Descemet’s membrane; St: stroma.

Examination of the TEM images showed that in the one-month samples from each strain group, the microvilli and organelles of the endothelial cell were abundant. The junction of the interlamination was tight. The thickness of the endothelium and Descemet’s membrane were uniform (Figure 3A,B), which were both of no significant differences between the two strain groups (p=0.067 in Table 3, p=0.378 in Table 4) was found. In the six-month samples of the SAM R1 strain, the microvilli and the organelles were decreasing, fewer vacuoles were observed in the interlamination (Figure 3C), and the thickness of the Descemet’s membrane was significantly increased (p=0.005 in Table 4) while there were no significant changes in the endothelium (p=0.078 in Table 3). In the six-month samples obtained from the SAM P8 strain, the microvilli and the organelles were significantly less well resolved, and much of the interstices were in the interlamination (Figure 3D). The thickness was significantly decreased in the endothelium (p=0.000 in Table 3) while the thickness was significantly increased in the Descemet’s membrane (p=0.000 in Table 4). Similarly, in the 12-month-old samples, the microvilli and the organelles were very few in number, the interstices were larger and more abundant in the interlamination, and the thickness of endothelium was uneven (Figure 3E,F), which was significantly thinner (p=0.023 and p=0.014, respectively in Table 3), while the thickness of Descemet’s membrane was significant increased when compared to the six-month old samples (p=0.000 and p=0.001, respectively in Table 4). However, no significant difference was found between the two strains (p=0.953 in Table 4).

**Table 3:** The thickness of the corneal endothelium lay in various ages of each experimental group.

**Groups**	**1 m (μm)**	**6 m (μm)**	**12 m (μm)**
R1	1.60±0.22 (n=40)	1.31±0.29 (n=36, p=0.078 versus 1 m)	0.93±0.46 (n=38, *p=0.023 versus 6 m)
P8	1.90±0.23 (n=36)	0.96±0.38 (n=38, *p=0.000 versus 1 m)	0.54±0.38 (n=38, *p=0.014 versus 6 m)
p value (R1 versus P8)	p=0.067	*p=0.031	*p=0.019

In the headings, m is the age in months. In the body of the table, n is the number of fields examined from the three individual specimens of each experimental group. The thickness of the corneal endothelium decreased significantly in the 12-month-old in the R1 strain group while it decreased significantly in both the 6-month to 12-month-old specimens in the P8 strain group. When comparing the two strain groups, the thicknesses of the corneal endothelium are significant thinner in 6-month- and 12-month-old specimens of the P8 strain than in that of the R1 strain group.

**Table 4:** The thickness of Descemet’s membrane of the cornea in various ages of each experimental group.

**Groups**	**1 m (μm)**	**6 m (μm)**	**12 m (μm)**
R1	1.03±0.16 (n=40)	1.58±0.25 (n=36, *p=0.005 versus 1 m)	2.90±0.41 (n=38, *p=0.000 versus 6 m)
P8	1.20±0.14 (n=36)	2.20±0.52 (n=38, *p=0.000 versus 1 m)	2.89±0.64 (n=38, *p=0.001 versus 6 m)
p value (R1 versus P8)	p=0.378	*p=0.002	p=0.953

In the headings, m is the age in months. In the body of the table, n is the number of fields examined from the three individual specimens of each experimental group. The thickness of Descemet’s membrane increased significantly in 6-month- and 12-month-old specimens of both R1 and P8 strain groups. When comparing between the two strain groups, the thicknesses of Descemet’s membrane are more significantly thickened in the six-month-old specimen of the P8 strain than in the six-month-old specimen of the R1 strain group while no significant differences were seen between the 12-month-old specimens of the two strain groups.

**Figure 4:**
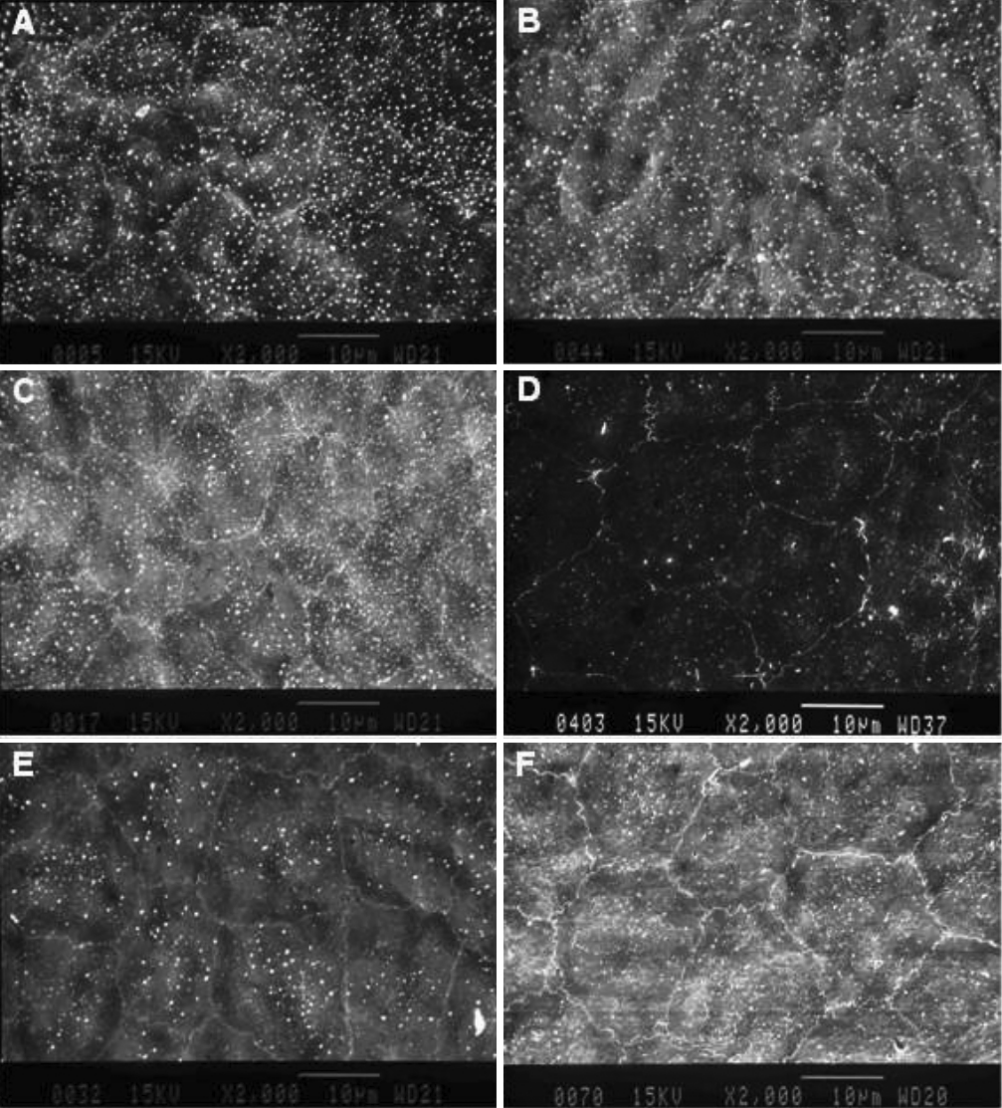
Analysis of scanning electron microscope imaging. Analysis of one-month-old specimens from the SAM R1 and the SAM P8 experimental groups (**A** and **B**, respectively) displayed the morphology of endothelial cells, which was hexagonal and uniform, with numerous microvilli located on the surface. Analysis of six-month-old specimens from the SAM R1 and the SAM P8 experimental groups (**C** and **D**, respectively) showed that the endothelial cells became larger and pantomorphic. Analysis of 12-month-old specimens from the SAM R1 and the SAM P8 experimental groups (**E** and **F**, respectively) showed more polygonal cells, and these cells also became larger. Scale bars: 10 μm.

In addition, examination of the SEM images showed that the one-month-old samples from each strain group had the hexagonal and uniform endothelial cell morphology with numerous microvilli located on the surface (Figure 4A,B). In the six-month-old samples from both strain experimental groups, the morphology of the endothelial cells was characterized as being larger and pantomorphic where the microvilli were fewer in number. Such changes were more significant in the SAM P8 strain (Figure 4C,D). In the 12-month-old samples from both strain groups, the cellular areas were even larger than that observed at six months, the microvilli were less numerous than that observed at six months, and a large number of pantomorphic cells were be observed (Figure 4E,F) with such changes more significant in the SAM P8 strain (Table 5).

**Table 5:** The percentage of hexagonal cells of the corneas in various ages of each experimental group.

**Groups**	**1 m (%)**	**6 m (%)**	**12 m (%)**
R1	84.11±11.17 (n=15)	75.33±8.40 (n=15, p=0.062 versus 1 m)	61.11±7.93 (n=15, *p=0.003 versus 6 m)
P8	86.78±8.27 (n=15)	65±10.10 (n=15, *p=0.000 versus 1 m)	51.67±11.86 (n=15, *p=0.006 versus 6 m)
p value (R1 versus P8)	p=0.564	*p=0.029	*p=0.045

In the headings, m is the age in months. In the body of the table, n is the number of fields examined from the three individual specimens of each experimental group. The percentage of hexagonal cells increased significantly in the 12-month-old specimen of the R1 strain group while the percentage increased significantly in both the 6-month- to 12-month-old specimens of the P8 strain group. When comparing the two strain groups, the percentage of hexagonal cells are significantly higher in 6-month- and 12-month-old specimens of the P8 strain than in the specimens of the R1 strain group of the same ages.

### Evaluation of the SA-β-Gal activity in corneal endothelial cells

**Figure 5:**
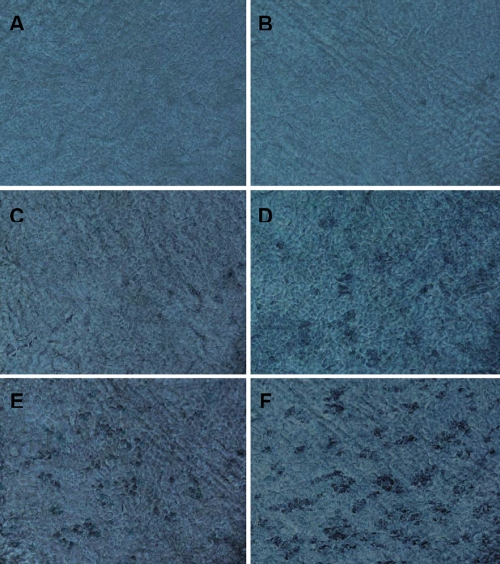
Staining of senescence-associated β-galactosidase activity in corneal endothelial cells of the SAM R1 and the SAM P8 strains at various ages. The positive stainings are shown in blue. The staining of the SAM R1 strain at one month, six months, and 12 months are shown (**A**, **C**, and **E**, respectively). The staining of the SAM P8 strain at one month, six months, and 12 months are also displayed (**B**, **D**,****and** F**, respectively). There were few to no positive stainings for SA-β-Gal activity in panels **A** and **B** while there were more multifocal and intense positive stainings in panels **C**, **D**, **E**, and **F**. Magnification: 400X.

**Figure 6:**
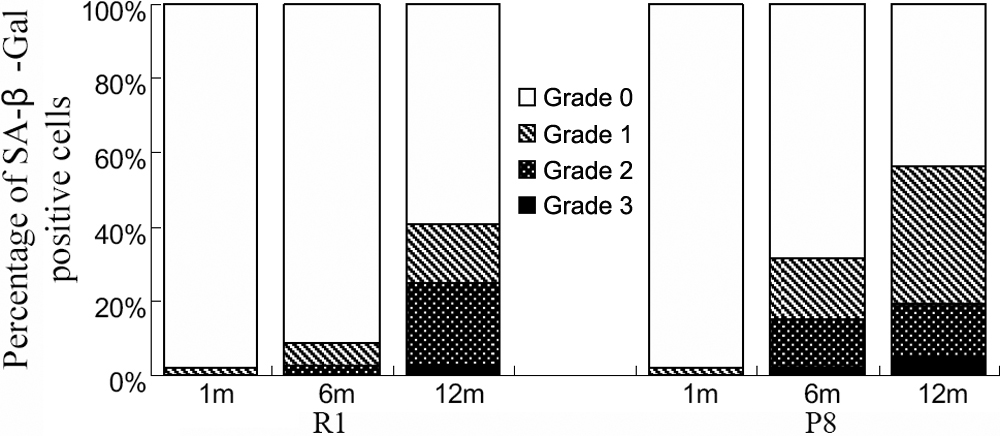
Average percentage of SA-β-Gal-positive cells at each grade in various experimental groups. The classification standard of SA-β-Gal activity is according to Tatsuya et al. [49]. This graph shows that few to no positive SA-β-Gal staining was observed in the endothelium in corneas from younger mice. In corneas from older mice, the percentage of CECs staining positive for SA-β-Gal was higher in the P8 strain than in the R1 strain.Statistical analysis is shown in Table 6.

SA-β-Gal staining, which served as a marker of cellular senescence, was performed and indicated very little activity, if any, in the one-month-old CECs from each experimental group (Figure 5A,B). However, analysis of the staining tendencies indicated that the expression increased with increasing age in the CECs from each strain group (Figure 5C-F) with such changes more significant in the SAM P8 strain when comparing the two strains (Table 6 and Figure 6).

**Table 6:** The percentage of SA-β-Gal-positive cells of the corneas in various ages of each experimental group.

**Groups**	**1 m (%)**	**6 m (%)**	**12 m (%)**
R1	1.87±1.64 (n=15)	8.93±8.65 (n=15, p=0.205 versus 1 m)	40.80±20.45 (n=15, *p=0.000 versus 6 m)
P8	2.13±1.06 (n=15)	31.40±22.75 (n=15, *p=0.000 versus 1 m)	56.33±13.32 (n=15, *p=0.000 versus 6 m)
p value (R1 versus P8)	p=0.959	*p=0.000	*p=0.003

In the headings, m is the age in months. In the body of the table, n is the number of fields examined from the three individual specimens of each experimental group. The percentage of SA-β-Gal-positive cells increased significantly in the 12-month-old specimen of the R1 strain group while the percentage increased significantly in both the 6-month- to 12-month-old specimens of the P8 strain group. When comparing the two strain groups, the percentage of SA-β-Gal-positive cells are significantly higher in both the 6-month- and 12-month-old specimens of the P8 strain than in specimens of the R1 strain group of the same ages.

### Qualitative immunohistochemical localization

**Figure 7:**
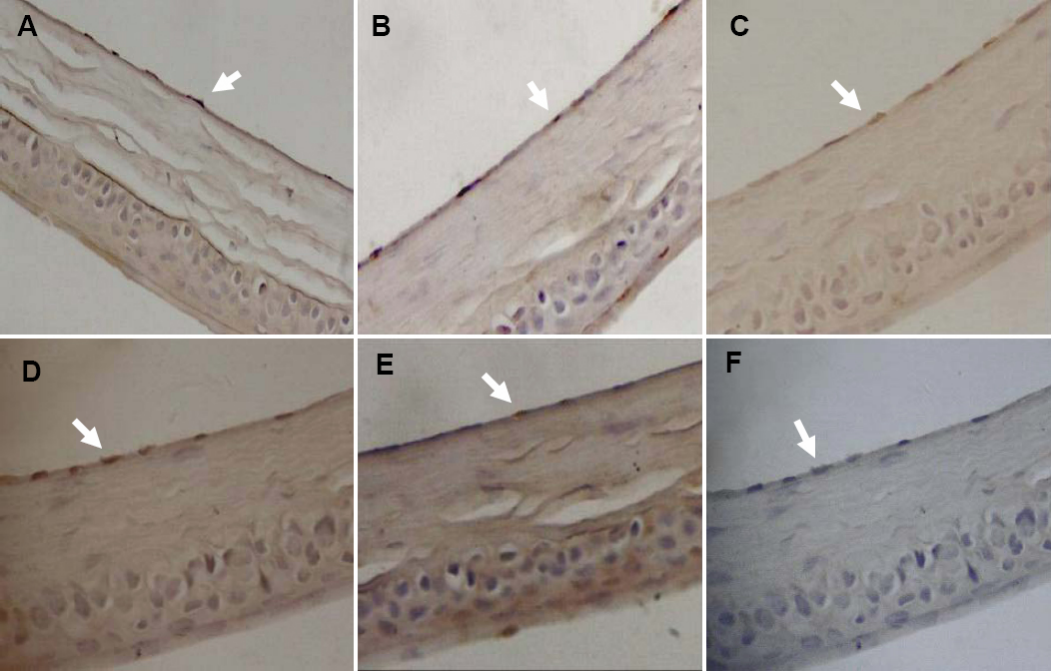
Immunohistochemical staining of p-ERK 1/2, p16^INK4a^, p19^ARF^, p21^WAF1/CIP1^, and p53 in corneal endothelial cells. The arrows indicate the positive staining in the endothelial nuclei at various ages of each experimental group **A**: p-ERK 1/2, SAM R1 strain at one month; **B**: p16^INK4a^, SAM P8 strain at one month; **C**: p19^ARF^, SAM P8 strain at 12 months; **D**: p21^WAF1/CIP1^, SAM P8 strain at six months; **E**: p53, SAM R1 strain at 12 months. **F**: The negative control incubated with PBS instead of a primary antibody, SAM P8 strain at six months. Magnification: 400X.

Immunohistochemical staining of p-ERK 1/2, p16^INK4a^, p19^ARF^, p21^WAF1/CIP1^, and p53 revealed that all of these proteins were expressed and were located in the nucleus of the CECs in each experimental groups (Figure 7).

### Real-time polymerase chain reaction of the senescence-related genes in corneal endothelial cells

**Figure 8:**
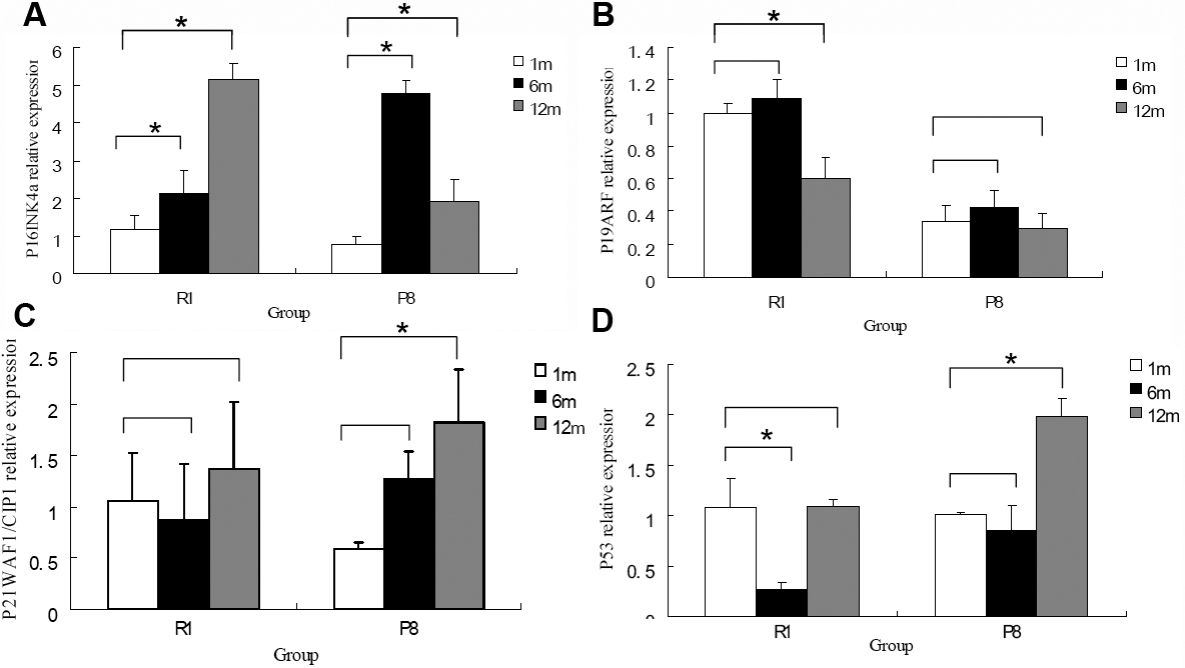
Quantitative real-time polymerase chain reaction analyses of mRNA expression of senescence-related genes after normalization to *GAPDH* in the corneal endothelial cells of SAM R1 and P8 strains at various ages. **A**: Quantitative comparison of the fold difference in the expression of *p16^INK4a^* mRNA is shown at various ages in each experimental group. The results indicate that the expression of *p16^INK4a^* was significantly increased from six months old to 12 months old in the SAM R1 strain experimental group (p=0.027 and p=0.000, respectively). In the SAM P8 strain experimental group, a significant increase was observed at six months (p=0.000 when compared to the one-month-old control group) and a significant decrease was observed at 12 months (p=0.000 when compared to the value at six months), although comparison between the 12-month- and 1-month-old values still resulted in a statistically significant increase (p=0.008). **B**: Quantitative comparison of the fold difference in the expression of *p19^ARF^* mRNA is shown at various ages. The results indicate that the expression of *p19^ARF^* mRNA decreased significantly at six months in the SAM R1 strain experimental group (p=0.000). **C**: Quantitative comparison of the fold difference in the expression of *p21^WAF1/CIP1^* mRNA is shown. The results indicated that the expression of *p21^WAF1/CIP1^* was significantly higher at 12 months in the SAM P8 strain experimental group (p=0.007). **D**: Quantitative comparison of the fold difference in the expression of *p53* mRNA is shown. The results indicate that the expression of *p53* decreased significantly at six months of age in the SAM R1 strain experimental group, although no significant difference was observed at 12 months of age (when compared to the value at one month; p=0.000, p=0.927, respectively). However, the expression of *p53* increased significantly at 12 months of age in the SAM P8 strain experimental group (p=0.000). There were three individual corneas used in each experimental group, and each cDNA sample had been detected three times. An asterisk marks a statistically significant difference (p<0.05).

The genes responsible for expressing p16^INK4a^, p19^ARF^, p21^WAF1/CIP1,^ and p53 proteins in CECs were detected quantitatively using real-time PCR. These data show that the relative expression of *p16^INK4a^* increased significantly in the SAM R1 strain with increasing age. Although we observed a significant increase of *p16^INK4a^* expression from six months of age to 12 months in the SAM P8 strain when compared to the value at one month, the expression of *p16^INK4a^* had decreased at 12 months when compared to the value at six months (Figure 8A). The relative expression of *p19^ARF^* decreased significantly in the 12-month-old samples from the SAM R1 strain. However, no significant differences in the expression level were observed with the SAM P8 strain as a function of increasing age (Figure 8B). In addition, the relative expression of *p21^WAF1/CIP1^* and *p53* mRNA increased significantly in the SAM P8 strain with an increase in age, but, again, a similar statistically significant increase was not observed for the SAM R1 strain (Figure 8C-E).

### Western blot analysis of the MAPK signaling pathway in the corneal endothelial cells as a function of age

**Figure 9:**
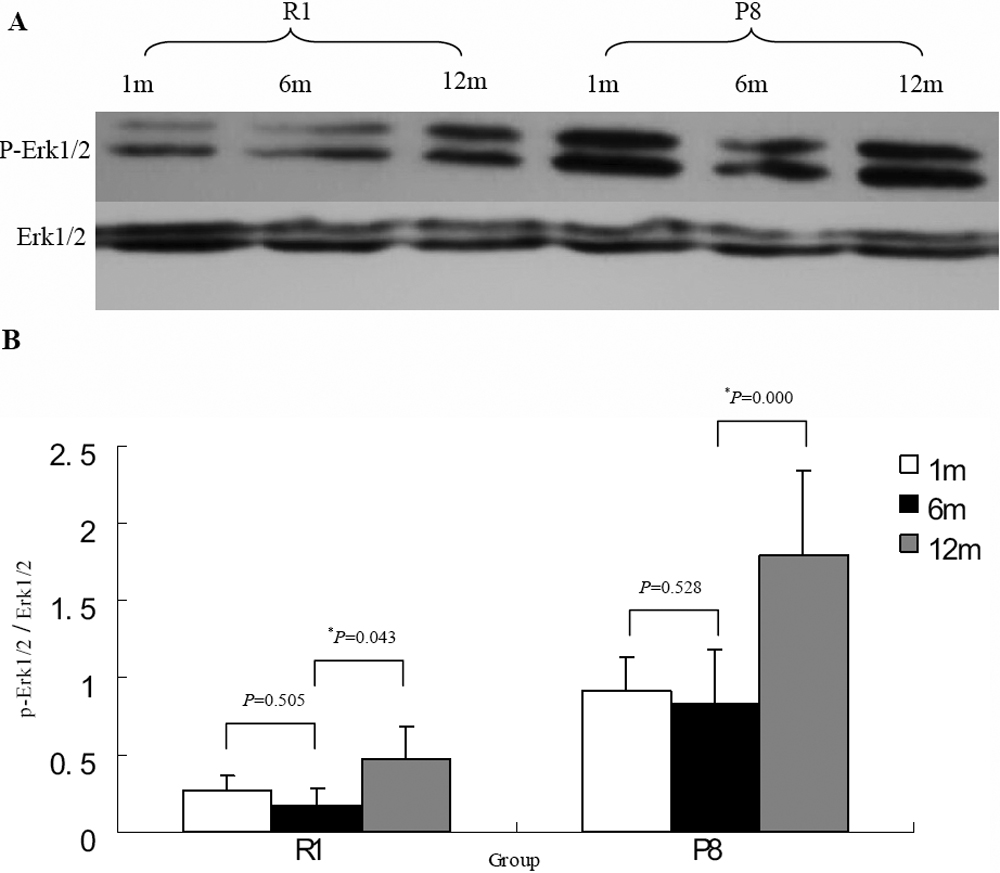
Western blot analyses of the expression levels of the p-ERK 1/2 protein in the corneal endothelial cells at various ages of the SAM R1 and SAM P8 strains. **A**: The expressions of p-Erk1/2 and Erk1/2 proteins in 1-month-, 6-month-, and 12-month-old specimens of the SAM-R1 and the SAM P8 strains are displayed. **B**: Quantitative comparison of the fold changes in the expression of p-Erk1/2 after normalization to the Erk1/2 protein is shown at various ages in each experimental group. The results show that p-ERK1/2 significantly increased at 12 months of age in both the R1 and P8 strains, which indicate that the activation level of Erk1/2 protein is much higher in the late senescent CECs. There were three individual corneas used in each experimental group, and each protein sample had been detected three times. An asterisk marks a statistically significant difference (p<0.05).

The Erk1/2 proteins are the key proteins of the MAPK signaling pathway, which is phosphorylated and subsequently activated. To further understand the activity state of the MAPK signal cascade as a function of the age of the CECs, we compared the expression levels of the p-ERK 1/2 proteins at various ages after birth. These data show that the expression of the p-ERK 1/2 protein increases at 12 months both in the SAM R1 and SAM P8 strains (Figure 9A,B).

## Discussion

HCECs, which are cells that play important roles in maintaining the structure and function of the cornea, are unable to proliferate in vivo. The quantity of HCECs decrease as aging progresses [32], and this tendency is coupled with senescence-induced changes in cellular morphology [2-4]. Previous studies have also reported atrophic and degenerative morphological characteristics in the endothelium of the corneal graft that had resulted in late endothelial failure (LEF) [5,6] or chronic corneal allograft dysfunction (CCAD) after penetrating keratoplasty (PK) [33]. This was evidenced by a decrease in endothelial cell counts, the observation of enlarged cells, irregularly shaped cells, cytoplasm loss, and the appearance of vacuoles and binuclear cells. Moreover, the ECD of the corneal grafts decreased at an accelerated rate when compared to healthy corneas [5,6]. All sorts of observations and evidence have indicated that such corneal diseases may be related to the accelerated senescence of the CECs, which has become a major cause of late transplant failure [6,31]. However, until now, the physiologic and pathological mechanisms of the HCEC senescence signaling pathways have not been elucidated.

Due to the lack of donors and to limitations in performing human in vivo studies, an appropriate animal model is needed to explore the process of senescence in CECs. The SAM has certain features that may make it a potentially attractive model for understanding cellular senescence and may in principle help in preventing several corneal diseases that are associated with senescence. Both the SAM R1 and SAM P8 strains were characterized by an age-dependent decrease in the ECD values that interestingly resemble the progression of HCECs with age. Light microscopy and electron microscopy analyses showed the degenerative and atrophic characteristics of these aforementioned strains, which are intriguingly similar to HCECs progressing through the senescence process, including a decrease in the number of microvilli and organelles, a thinning of the endothelium, a thickening of the Descemet’s membrane, an increase in the number of vacuoles and pantomorphic cells, and an increase in the size of the cells. These specific characteristics were more significantly altered in the SAM P8 strain than in the SAM R1 strain. In addition, the results from SA-β-Gal staining analysis, which is known to represent a marker of cellular senescence, indicated that the number of senescent cells increased in the CECs of these two mouse strains with an increase in age. However, the characteristic changes were far more obvious in the SAM P8 strain. All of the above results demonstrate that the CECs of the SAM-R1 and SAM P8 strains enter senescence as they age, and moreover, the aging process progresses more rapidly in the CECs of the SAM P8 strain. However, it is still unclear whether the vascular membrane hyperplasia in CECs of 12-month-old SAM P8 mice is due to senescence of CECs or is just an accident. More research is required to detect this phenomenon.

Based on the above results, we made further investigations on corneal endothelial cellular senescence in the SAM strains from the molecular level. Many studies have reported the importance of senescence-related genes such as *p16^INK4a^*, *p19^ARF^*, *p21^WAF1/CIP1^*, and *p53* in cellular senescence. They can inhibit cell cycle progression and maintain the G_1_ phase arrest of cells [34]. They are always looked as biological markers of cell senescence for their accumulation and high expression in the aged cells [35,36]. Some environmental stress can also lead to the overexpression of these genes, which may induce premature cells [37-40]. There were some reports on the importance of these genes in senescence of CECs in vitro, but their expression may be influenced by passage, culture environment, injury, or other factors. Therefore, the expression of these genes in vivo may not directly be inferred from these in vitro findings. Although the proliferative ability of CECs may be different between different species, the ECD of rodents decrease with age as it does in human [41,42]. There is no limitation on number of animals used, and dynamic observation and intervention can be made throughout the course of senescence. Therefore, this mouse model is quite important and irreplaceable in the studies on aging mechanisms of CECs in vivo.

In this study, immunohistochemical and real-time PCR analyses not only confirmed but also allowed for the comparison of the relative expression levels of *p16^INK4a^*, *p19^ARF^*, *p21^WAF1/CIP1^*, and *p53* in CECs. An age-dependent increase in *p16^INK4a^* expression was observed in the SAM R1 strain and in the early stages of senescence in the SAM P8 strain. However, an age-dependent increase in the expression of *p21^WAF1/CIP1^* and *p53* was only observed in the SAM P8 strain. Thus, the p16^INK4a^ and the p53/p21^WAF1/CIP1^ pathways should be related to the in vivo process of aging in the CECs of SAM. However, the reasons for the differential expression of these signaling pathway components in each strain may be related to the extent to which aging has progressed and the relative rate of the different signaling cascades. The SAM R1 strain is characterized as having a normal aging process in mice such that 12 months are equivalent to roughly two-third of its entire life span while the SAM P8 strain represents an accelerated senescence mouse model where six months of age is equivalent to approximately half of its entire life span. The old donors studied in a report by Song et al. [8] were between 50 and 65 years old, which was equivalent to approximately half to two-third of a typical human life span. Thus, the results, which indicated an age-dependent increase in the expression of *p16^INK4a^* at 6 and 12 months of age for the SAM R1 strain and at six months of age for the SAM P8 strain, were similar to the aforementioned study in HCECs [8]. These results have important implications for our hypothesis regarding the aging program in CECs, specifically that the p16^INK4a^ pathway may play an important role in early senescence. We have also observed an age-dependent increase in the expression levels of *p53* and *p21^WAF1/CIP1^* in CECs of the SAM P8 strain. However, there were no such differences observed in the characteristics of HCECs [8]. Species-related differences may be one of the reasons for this observation. However, we can still make the assumption that the p53/p21^WAF1/CIP1^ signaling pathway may cooperate to promote CECs senescence, although this would primarily have an effect in the late stages of aging. Considering the SAM P8 strain represents a senescence-accelerated mouse, we also deduce that the activation of the p53/p21^WAF1/CIP1^ signaling pathway may promote an accelerated senescence in CECs during some pathological processes.

Interestingly, both *p16^INK4a^* and *p19^ARF^* are located at the INK4a locus (one of the most frequently disrupted tumor suppressor loci in human cancer) [43], and they have both been shown to act as effectors of senescence in cultured cells [44]. In addition, their expression levels increase in numerous tissues with increased age [45,46]. However, in our study, the expression of *p19^ARF^* did not increase with increased age in either of the two strains and was even shown to decrease in the SAM R1 strain at 12 months. Until now, there have not been any studies that have probed the age-dependent expression of *p19^ARF^* in CECs either in vivo or ex vivo. However, in the skeletal muscle and fat tissue of mice lacking BubR1 (an essential spindle checkpoint protein conserved among higher eukaryotic organisms), the inactivation of *p16^INK4a^* attenuates both cellular senescence and premature aging, and conversely, *p19^ARF^* inactivation exacerbates cellular senescence. These observations demonstrate that p16^INK4a^ is an effector and that p19^ARF^ is an attenuator of cellular senescence in these tissues [47]. However, information concerning whether or not p19^ARF^ has the same effect on senescence in CECs remains unclear. Therefore, further investigation that specifically compare samples from human and SAM strains comprising a broader range of ages are necessary to accurately characterize the senescence signaling pathway in CECs.

Most studies have indicated that the MAPK signaling pathway in mammalian systems have shown a transmission of mitogenic signals that promotes proliferation and differentiation. However, studies that are more recent have indicated that the MAPK signaling cascade is initially mitogenic but eventually induces senescence via the activation of the p53 and p16^INK4a^ tumor suppressor in primary cells [48]. These studies found that activated MEK (a component of the MAPK signaling cascade) permanently arrests the primary murine fibroblasts and initiates uncontrolled mitogenesis and transformation in cells lacking either p53 or INK4a. These studies indicated that the cell cycle arrest induced by the MAPK signaling cascade is permanent and reflects a bona fide cellular senescence and not an unusual form of quiescence or differentiation. In our study, we found that the expression levels of the p-ERK 1/2 proteins (a component of the MAPK cascade that had been activated by phosphorylation) were significantly higher in CECs of older mice compared with one- and six-month-old mice from each experimental group. However, it has yet to be determined whether the activated and upregulated states of Erk1/2 induce the senescence process of CECs.

In summary, the SAM R1 and the SAM P8 strains are sufficient models for the study of corneal endothelial cellular senescence in vivo. The CEC senescence program progresses more rapidly in the SAM P8 strain than in the SAM R1 strain. The p16^INK4a^ signaling pathway may induce CEC senescence in the SAM R1 strain and in early stages of senescence in the SAM P8 strain. However, the p53/p21^WAF1/CIP1^ signaling pathway may induce CEC senescence in the SAM P8 strain. We conclude from analysis of these results that the p16^INK4a^ signaling pathway may play a key role in the early process of senescence in CECs and that the p53/p21^WAF1/CIP1^ signaling pathway may have its principle effect in the late stages of senescence in CECs. However, it has yet to be determined whether MAPK signaling pathway plays a role in the senescence process of CECs. Further investigation is needed.
